# Mathematical Methods in Biomedical Imaging 2014

**DOI:** 10.1155/2014/156494

**Published:** 2014-12-28

**Authors:** Peng Feng, Kumar Durai, Fenglin Liu, Xiaobo Qu

**Affiliations:** ^1^Key Laboratory of Opto-Electronics Technology & System, Chongqing University, Ministry of Education, Chongqing 400044, China; ^2^Electronics and Communication Engineering Department, Periyar Maniammai University, Vallam, Thanjavur 613403, India; ^3^Biomedical Imaging Cluster, Department of Biomedical Engineering, Rensselaer Polytechnic Institute, Troy, NY 12180, USA; ^4^Research Center of Magnetic Resonance and Medical Imaging, Department of Electronic Science, Xiamen University, Xiamen 361005, China

The last quarter century has witnessed major advancements that have brought biomedical imaging to a paramount status in life sciences. Generally speaking, the scope of biomedical imaging covers data acquisition, image reconstruction, and image analysis, involving theories, methods, systems, and applications. While many kinds of imaging modalities, such as X-ray computed tomography (CT) and magnetic resonance imaging (MRI), become increasingly sophisticated, the mathematical methods involved in these modalities play more and more critical roles in further improving their performance in anatomical, functional, cellular, and molecular applications. The overall goal of this issue is to promote research and development of biomedical imaging by publishing high-quality research articles in this rapidly growing interdisciplinary field. Due to the time limit, this special issue mainly focused on 4 kinds of biomedical imaging modalities: CT, MRI, ultrasound, and fluorescence imaging; several biomedical image processing methods were also involved. Each paper published in this special issue was reviewed by at least two reviewers and revised according to reviewer's comments.

For CT imaging, A. Cai et al. developed an efficient iterative image reconstruction (IIR) algorithm, using cone beam CT reconstruction that is based on total-variation (TV) minimization to overcome the computational complexity of IIR scheme in cone beam CT reconstruction; L.-z. Deng et al. proposed a hybrid reconstruction method combining TV and nonaliasing contourlet transform (NACT) and using the Split-Bregman method to solve the optimization problem. This algorithm utilized the geometrical information of CT image and got a sparser representation compared with wavelet and gradient operator.

For MRI imaging, in order to reduce time consuming in MRI image reconstruction, Q. Li et al. proposed a parallel computing method which was based on a novel patch-based nonlocal operator (PANO). Simulation results demonstrated that this method can accelerate PANO-based MRI reconstruction several times compared with original one. W. He et al. introduced a direct nonconvex *L*_*p*_ norm algorithm for MRI phase unwrapping which leaded to faithful phase correction. Also analytical high order tensor decomposition was introduced into crossing fibers detection in diffusion MRI by T. Megherbi et al., which provided a better angular resolution and accuracy than the classical maxima localization method.

For biomedical image processing, R. Jaramillo et al. used a wavelet domain filter to improve the performance of the Prony method. In this work, MRI images were considered to be affected only by Rician noise, and a new wavelet domain bilateral filtering process was implemented to reduce the noise in the images. X. Wang et al. proposed a model that allows robustness to noise as well for handling the intensity inhomogeneity and weak boundary problems in medical image segmentation using region-scalable discriminant and fitting energy for image segmentation. Also, a region-based active contour model which introduced a total energy as a penalty function in medical image segmentation is proposed by T. Liu et al. Another active contours based segmentation method was proposed by F. Akram et al. for intensity inhomogeneous MRI images to enable boundaries detection of the homogenous regions.

Not only CT and MRI but also other medical imaging modalities, such as ultrasound and fluorescence imaging, are included in this special issue. S.-K. Ueng et al. designed a special filter aiming at suppressing speckles and enhancing features in the ultrasound images. In this method, diffusion tensor of intensity at each pixel was represented in the form of a Hessian matrix which was used to compute eigen values at each pixel. The eigen values were used in detection and classifying the underlying structure and a refinement strategy was followed to improve the classification. Then, based on the computed structure types, feasible filters were selected from a filter pool to suppress speckles and enhance features. X. Lin et al. used three-dimensional fluorescent spectra imaging to investigate whether and how Tubeimoside 1 (TBMS 1) can affect HepG2 cells, which indicated that fluorescent spectra method is a promising substitute for flow cytometry in cancer research. A computational model to estimate fluence rate for a biological medium with inclusion was developed by M. Gantri. In this work, the entire setting of the medium was treated to have spatially and stochastically varying refractive index to match practical applications and Legendre integral transform technique is incorporated to solve the radiative transfer equation.

These papers represent an insightful observation into the state of the art, as well as future topics in this biomedical imaging field. We hope that this special issue would attract a wide attention of the peers and provide a chance to share the latest research work.

*Peng Feng* *Peng Feng* *Kumar Durai* *Kumar Durai* *Fenglin Liu* *Fenglin Liu* *Xiaobo Qu* *Xiaobo Qu*

